# The Down Syndrome Profile Emerges Gradually Across Early Development

**DOI:** 10.1111/jar.70218

**Published:** 2026-07-19

**Authors:** Hana D'Souza, Annette Karmiloff‐Smith, Denis Mareschal, Michael S. C. Thomas

**Affiliations:** ^1^ Centre for Human Developmental Science, School of Psychology Cardiff University Cardiff UK; ^2^ Centre for Brain and Cognitive Development Birkbeck, University of London London UK; ^3^ The LonDownS Consortium London UK

**Keywords:** cross‐sectional developmental trajectories, Down syndrome, early development, individual differences, Mullen Scales of Early Learning, profile, strengths and weaknesses

## Abstract

**Background and Aims:**

Down syndrome (DS) is associated with intellectual disability, with particular difficulties in expressive language and gross motor abilities, and relative strengths in receptive language. Here, we examine how this profile arises over the first five years of life.

**Methods:**

A total of 104 children with DS (6–63 months) participated in a standardised developmental assessment (Mullen Scales of Early Learning; MSEL). Developmental trajectories were analysed cross‐sectionally and validated with a longitudinal subsample.

**Results:**

The trajectories gradually diverged from typical development, with an uneven pattern across domains. As children with DS get older, their gross motor difficulties persist, their expressive language becomes a relative weakness, and their receptive language becomes an area of emerging relative strength. Longitudinal data revealed limited stability of individual differences except for visual reception.

**Conclusion:**

Understanding how the DS profile emerges, as well as how stable individual differences are, presents important steps towards tailored support.

## Summary

- Children with Down syndrome show slower, uneven development, diverging from the typical developmental trajectory over the early years.
- Early gross motor difficulties persist, expressive language becomes a relative weakness, and receptive language becomes an area of emerging relative strength.
- With the exception of visual reception, there was a lack of stability of individual differences across time, and thus ongoing monitoring and support are necessary.

**Abbreviations**
AE: age equivalent
DQ: developmental quotient
DS: Down syndrome
MSEL: Mullen Scales of Early Learning
TD: typically developing
VABS: Vineland Adaptive Behavior Scales

## 1 Introduction

Down syndrome (DS) results from the presence of an additional copy of chromosome 21. With an occurrence of approximately 1 in 800 births worldwide (Bull 2020), DS is the most common known genetic cause of intellectual disability. Although large individual differences exist in the DS phenotype and severity of associated difficulties (Dykens and Hodapp 2001; Karmiloff‐Smith et al. 2016), a general profile has been characterised in older children and adults with DS. In addition to intellectual disability often falling within the mild to severe range (Chapman and Hesketh 2000), the profile includes relative strengths in receptive language, visuospatial processing, and some aspects of social functioning; and particular weaknesses in expressive language, motor ability, auditory processing, and verbal short‐term memory (e.g., Daunhauer and Fidler 2011; Jarrold et al. 1999; Jarrold and Baddeley 1997; Miller 1999; Miller and Leddy 1999; Pitcairn and Wishart 1994; Vicari 2006; Vicari and Carlesimo 2006; Wishart and Johnston 1990; for recent reviews, see Esbensen et al. 2024; Hamadelseed et al. 2023).

There is increasing evidence that the DS profile—with its loose assemblage of relative strengths and weaknesses—is not the direct result of genes but their probabilistic atypical modulation of complex developmental processes (see D'Souza et al. 2017; D'Souza and D'Souza 2022, 2024, for discussion). Therefore, in order to understand the profile, it is important to examine how it gradually emerges over developmental time (D'Souza and D'Souza 2022; D'Souza and Karmiloff‐Smith 2017; Karmiloff‐Smith 1998). This type of research is possible in DS because, unlike many other neurodevelopmental conditions, DS is often diagnosed pre‐ or perinatally (Hill et al. 2017; Thomas et al. 2020). Indeed, interest in early development in DS is ever growing (e.g., D'Souza, D'Souza, Jones, and Karmiloff‐Smith 2020; D'Souza and D'Souza 2022; Fidler, Schworer, Prince, et al. 2019; Fidler, Schworer, Will, et al. 2019; Onnivello, Schworer, Daunhauer, and Fidler 2023; Onnivello, Schworer, Prince, et al. 2023; Seager et al. 2022). However, only a small number of studies comprehensively mapped how the DS profile unfolds over early development. Here we specifically focus on work that directly compares a broad range of domains across the early years (0–5 years). Older studies on this topic (e.g., Carr 1970; Cunningham 1996; Shepperdson 1995) provided valuable insights but reflect very different healthcare contexts and developmental expectations than those experienced by children with DS born more recently (Coppus 2013). More recent studies (post‐2000) tend to fall into two categories: those with wide age ranges but relatively shallow phenotyping (assessing only a few domains, often via parental report; e.g., Dykens et al. 2006; Van Duijn et al. 2010; Will et al. 2018), and those with narrower age ranges but more in‐depth phenotyping (covering multiple domains, typically through experimenter‐led testing; e.g., Fidler et al. 2006; Pezzuti et al. 2023).

Belonging to the first type of studies, Dykens et al. (2006) investigated profiles and cross‐sectional developmental trajectories of adaptive behaviour in 80 children with DS from one to 11.5 years using a parent‐report measure—the Vineland Adaptive Behavior Scales (VABS; Sparrow et al. 1984). Children with DS were reported to show a significant weakness in communication relative to daily living skills and socialisation skills. Expressive language was significantly weaker than receptive language, especially when the overall communicative levels of children were above 24 months (age equivalent). However, the motor domain was not investigated in this study, which limits a full understanding of the profile. This domain was included in a study by Will et al. (2018) of 64 5‐ to 45‐month‐olds with DS, alongside communication, daily living, and socialisation domains (measured using the VABS). A developmental delay in the DS group was already apparent by the end of the first year of life, and the delay increased over developmental time. The greatest divergence between the DS group and the chronological age‐matched typically developing (TD) group was reported for motor and communication abilities. However, more detailed distinctions within these domains (fine vs. gross motor abilities; receptive vs. expressive language abilities) were not investigated. Again, this limits our understanding of the DS profile.

Belonging to the second type of studies, which employ more in‐depth phenotyping but with a limited age range, Fidler et al. (2006) tested 18 2‐ and 3‐year‐olds with DS, 19 mental age‐matched 2‐ and 3‐year‐olds with mixed developmental disabilities, and 24 mental age‐matched TD children. No clear differences were found between these groups on an experimenter‐led standardised test, the Mullen Scales of Early Learning (MSEL; Mullen 1995). This suggests that the DS profile of strengths and difficulties had yet to emerge. However, comparisons of different domains measured using the MSEL (gross motor, fine motor, visual reception, receptive language, expressive language) within the DS group suggested emerging areas of relative weakness in expressive language and gross motor abilities. Therefore, an emerging profile of strengths and difficulties in DS can already be detected at 2–3 years of age. Looking at a slightly wider age range (1–3 years of age), Pezzuti et al. (2023) used an Italian adaptation of the Bayley Scales of Infant and Toddler Development (BSID‐III; Bayley 2006; adapted by Ferri et al. 2015) with 144 toddlers with DS. Overall, receptive and expressive language were described as relative strengths in this age group. When participants were split into the younger (1–2 years) and older (2–3 years) subsamples, fine motor (but not gross motor) abilities showed an increase in standard scores with age. However, a notable decrease in standard scores was reported for expressive language. Taken together, the DS profile seems to emerge around the age of two. This is consistent with findings that, from around this age, early abilities predict performance later in life in children with DS (Carr 1995; Cunningham 1996; Marchal et al. 2016).

In summary, the previous literature provides some clues about the emerging DS profile, but a more comprehensive study that would examine a large sample of young children with DS across early years, and with more in‐depth phenotyping, is needed. This is the aim of the current study. Firstly, employing the experimenter‐led MSEL with over a hundred children with DS from 6 to 63 months of age enables us to build cross‐sectional developmental trajectories (Thomas et al. 2009) to understand how the DS profile of relative strengths and weaknesses emerges and changes over developmental time. Secondly, utilising longitudinal data gathered from a subset of the children enables us to validate the cross‐sectional developmental trajectories and investigate the stability of individual differences across developmental time.

## 2 Methods

### 2.1 Participants

One hundred and four children with DS (45 females) between 6.13 and 63.40 months (*Mdn* = 25.78, *IQR* = 22.05) participated in this study (for participant characteristics, see Table 1). Three children were excluded from the analysis due to reaching the ceiling on one of the scales of the MSEL (gross motor: *n* = 2 [56.80 and 58.20 months], visual reception: *n* = 1 [62.37 months]; for details on the MSEL, see Section 2.2). One participant (47.30 months) did not complete the visual reception scale but was included in domain‐level analyses for the other scales to maximise the sample size. The participants were recruited via existing participant databases and support groups in the UK and were part of the LonDownS cohort (for detailed demographics and health information, see Startin et al. 2020). Ethical approval was obtained from the North West Wales National Health Service (NHS) Research Ethics Committee (13/WA/0194) and from the Birkbeck Psychological Sciences Ethics Committee (121373). Prior to testing, informed consent was obtained from parents. Participants were given a small gift (e.g., a T‐shirt) in return for their participation.

**TABLE 1:** Participant characteristics.

		Sample		Comparison
		Cross‐sectional	Longitudinal (at Time 1)	
Number		104	43	NA
Chronological age (months)		6.13–63.40 *Mdn* = 25.78, *IQR* = 22.05	11.93–44.57 *Mdn* = 20.37, *IQR* = 14.03	*U* = 1668.50, *p* = 0.016*
Sex	Female	45 (43.3%)	15 (34.9%)	*χ* ^*2*^(1) = 0.89, *p* = 0.364
	Male	59 (56.7%)	28 (65.1%)	
Ethnicity	White	84 (80.8%)	38 (88.4%)	Fisher's exact test = 1.94 *p* = 0.811
	Asian	6 (5.8%)	3 (7.0%)	
	Black	5 (4.8%)	1 (2.3%)	
	Mixed	7 (6.7%)	1 (2.3%)	
	Other	1 (1.0%)	0 (0.0%)	
	Missing	1 (1.0%)	0 (0.0%)	NA
Parental occupation	Managers, directors and senior officials	26 (25.0%)	11 (25.6%)	Fisher's exact test = 5.70 *p* = 0.582
	Professional occupations	40 (38.5%)	13 (30.2%)	
	Associate professional and technical occupations	15 (14.4%)	5 (11.6%)	
	Administrative and secretarial occupations	4 (3.8%)	4 (9.3%)	
	Skilled trades occupations	3 (2.9%)	2 (4.7%)	
	Caring, leisure and other service occupations	3 (2.9%)	3 (7.0%)	
	Sales and customer service occupations	2 (1.9%)	2 (4.7%)	
	Process, plant and machine operatives	1 (1.0%)	0 (0.0%)	
	Elementary occupations	0 (0.0%)	0 (0.0%)	
	Missing	10 (9.6%)	3 (7.0%)	NA
Congenital heart defects	Total	53 (51.0%)	20 (46.5%)	*χ* ^*2*^(1) = 0.10, *p* = 0.852
	Known AVSD	40 (38.5%)	13 (30.2%)	*χ* ^*2*^(1) = 0.68, *p* = 0.446
	Surgery	20 (19.2%)	6 (14.0%)	*χ* ^*2*^(1) = 0.47, *p* = 0.632
	Missing	4 (3.8%)	3 (7.0%)	NA
Reflux	Total	33 (31.7%)	12 (27.9%)	*χ* ^*2*^(1) = 0.12, *p* = 0.842
	Missing	4 (3.8%)	3 (7.0%)	NA
Vision & hearing	Vision impairments	23 (22.1%)	8 (18.6%)	*χ* ^*2*^(1) = 0.15, *p* = 0.823
	Hearing impairments	29 (27.9%)	11 (25.6%)	*χ* ^*2*^(1) = 0.03, *p* = 1.000
	Otitis media with effusion (glue ear)	56 (53.8%)	21 (48.8%)	*χ* ^*2*^(1) = 0.14, *p* = 0.851
	Missing	4 (3.8%)	3 (7.0%)	NA

*Note:* A subset of the cross‐sectional sample (“Cross‐sectional”) was followed up longitudinally. “Longitudinal (at Time 1)” corresponds to values associated with the longitudinal subsample at the first timepoint, to allow for direct comparison of whether it differs from the larger cross‐sectional sample. As a *U*‐test was carried out due to non‐normal distribution, *Mdn* and *IQR* rather than *M* and *SD* are reported here for chronological age. For details on how variables in this table were collected, see Startin et al. (2020). The table includes health comorbidities which show prevalence above 10% in young children with DS (Startin et al. 2020). *p < 0.050.

Abbreviations: AVSD, atrioventricular septal defect; NA, not applicable.

**FIGURE 1:**
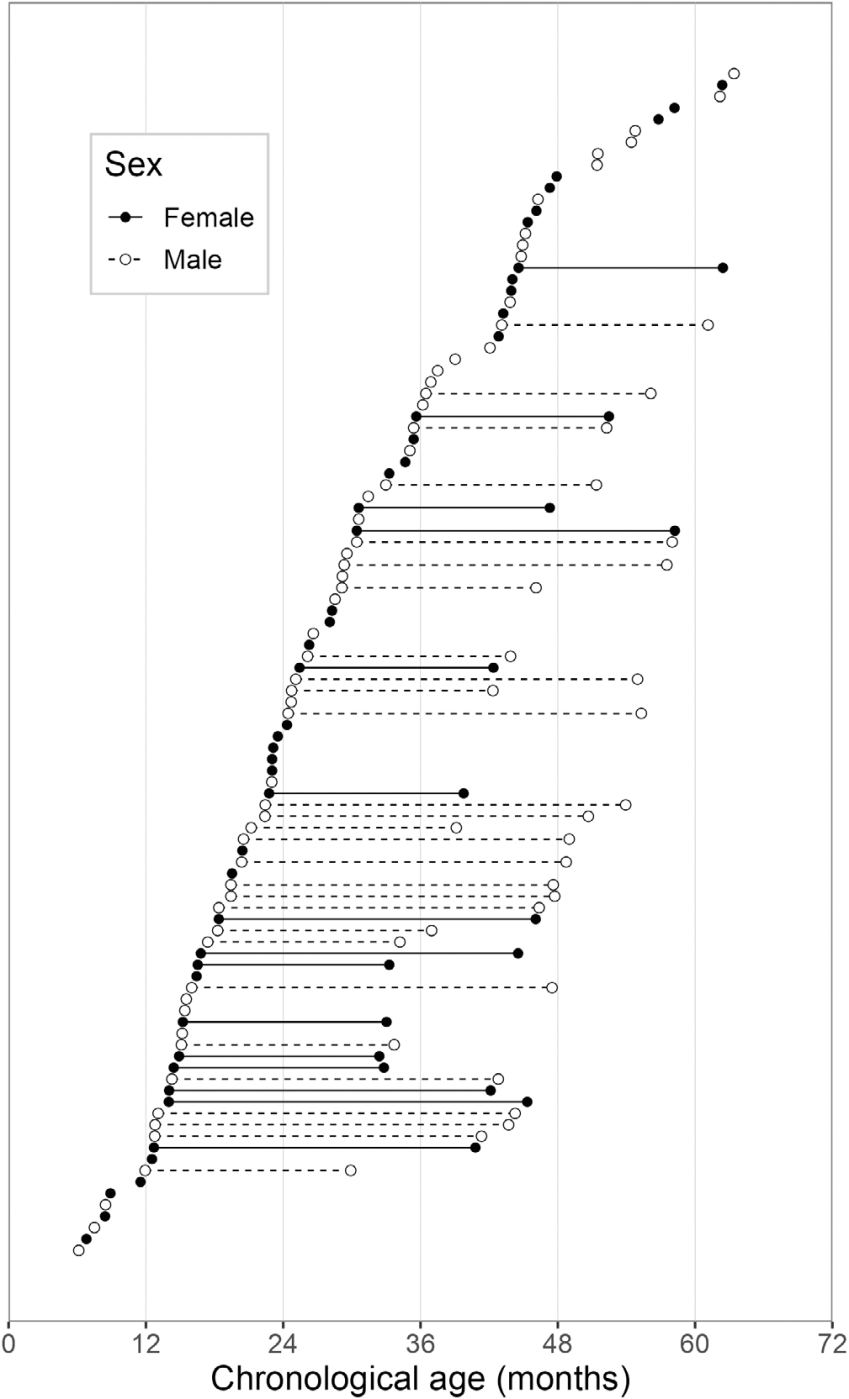
Age of participants at time of testing. Each testing session is represented by a circle; each of the 104 participants is shown in a different row (full circles and full lines = females, empty circles and dashed lines = males). For participants with two time points, Time 1 and Time 2 are connected by a straight line. Note the relatively even spacing of participants across the age range, with the two different lengths of longitudinal follow‐up—one ~18 months long, the other ~30 months long.

Out of this cross‐sectional sample, 43 children (15 females) were tested a second time (Time 2) after around 18 months (*n* = 21, *Mdn* = 17.74, *IQR* = 1.30, range = 16.70–19.66 months) or 30 months (*n* = 22, *Mdn* = 28.33, *IQR* = 2.80, range = 27.57–31.50 months) to allow for staggered analysis (Figure 1). Six children in this longitudinal sample reached ceiling on one or more scales of the MSEL at Time 2 (gross motor: *n* = 2 [47.50 and 47.60 months], gross motor + visual reception: *n* = 2 [50.67 and 62.43 months], visual reception + receptive language: *n* = 2 [52.27 and 61.13 months]) and thus were excluded from the longitudinal analysis. The cross‐sectional sample did not differ from the longitudinal subsample at Time 1 on any of the reported characteristics (see Table 1), except, as we would expect from the nature of the design, chronological age.

### 2.2 Procedure and Materials

The *Mullen Scales of Early Learning* (MSEL; Mullen 1995) was administered by an experimenter during the lab visit. Given the young age of the children in this study, there was variability in whether any vision or hearing impairments had been corrected, as well as in how consistently the children tolerated such corrections (e.g., some children removed their glasses after a short period). However, the experimenter ascertained that each child could see and hear the test items as required, both through direct observation and by consulting with parents.

The MSEL is a standardised assessment which comprises five domains: (1) *gross motor* (central motor control and mobility in supine, prone, sitting, and fully upright positions); (2) *fine motor* (visually‐directed motor planning, object manipulation, visual discrimination [motor planning], and motor control); (3) *visual reception* (visual perceptual ability, spatial awareness, and visual memory); (4) *receptive language* (auditory comprehension, auditory memory, and the ability to process linguistic input); and (5) *expressive language* (the ability to use sounds and language productively). These yield scores for each domain. The MSEL is standardised for TD children between 0 and 68 months (from 0 to 33 months for the gross motor domain). This test has high internal consistency and test–retest reliability (Mullen 1995; see also Bishop et al. 2011, for estimates of convergent validity).

Following the previous literature on DS (Dykens et al. 2006; Fidler et al. 2006; Marchal et al. 2016), we use age equivalent (AE) and developmental quotient (DQ) scores as opposed to standard scores in data analysis in order to compare across domains whilst maximising inclusion. Standard scores on standardised tests are often at floor in populations with intellectual disability, which obscures individual variability and fails to capture meaningful developmental progress. AE scores were derived from raw scores and transformed based on normative samples. Although AE scores are generally less useful than standard scores for comparing between individuals (Maloney and Larrivee 2007), they can nevertheless reflect developmental progress (see Toffalini et al. 2019, for discussion). DQ was calculated as AE divided by chronological age, multiplied by 100 ([AE/chronological age]*100). Thus, DQ is a measure of how close the child is to the level expected of a TD child of the same chronological age.

### 2.3 Cross‐Sectional Developmental Trajectory Analysis

In order to investigate changes in MSEL scores across chronological age, we employed a *cross‐sectional developmental trajectory analysis* (Thomas et al. 2009), which is well established in the field of neurodevelopmental conditions (e.g., D'Souza, Mason, Mok, et al. 2020; Jarrold et al. 2007; Purser et al. 2015; Thomas et al. 2011). In this analysis, development is modelled by explicitly fitting linear trajectories to cross‐sectional data and comparing them across MSEL scales. The goal of this analysis is to generate predictions about what the longitudinal trajectories should look like, which are then tested by the longitudinal follow‐up. This approach places developmental processes at the heart of explanations of neurodevelopmental conditions (Karmiloff‐Smith 1998).

The cross‐sectional developmental trajectory analysis is built on a *modified* Analysis of Covariance (ANCOVA). A standard ANCOVA can be used to test for differences between the *means* of different MSEL scales while “controlling” for chronological age (the covariate). Thus, after “accounting for” the different ages of the children in the sample, performance on each scale would be represented by a single number. In contrast, the cross‐sectional developmental trajectory analysis puts the focus on change across age by examining the effect of chronological age (the covariate) itself. It evaluates the differences between the regression lines which depict the developmental trajectories of MSEL scores across chronological age in different domains. Thus, two numbers represent cross‐sectional developmental trajectories—the *intercept* and *gradient*. The intercept represents the initial level of performance. The gradient represents the rate at which performance changes (increases or decreases) with age. The use of intercepts and gradients allows us to look beyond mere differences between *means* (i.e., a standard ANCOVA, which can only inform us of *whether* performance differs across scales or not) and provides insight into *how* scales differ across developmental time.

In this study, cross‐sectional developmental trajectories were initially constructed separately for each MSEL scale by conducting a series of regression analyses to examine whether chronological age was related to performance on a particular MSEL scale. In order to validate these cross‐sectional developmental trajectories, linear generalized estimating equations (GEE) for each MSEL scale were run with an unstructured correlation matrix on the longitudinal data. Subsequently, the cross‐sectional developmental trajectories were compared across scales. This made it possible to evaluate whether the developmental trajectories of the MSEL scales differed from each other in terms of their gradients and/or intercepts. To compare pairs of trajectories, a fully‐factorial ANCOVA was employed to test for a main effect of chronological age, MSEL scales, and the interaction between chronological age and MSEL scales. If the main effect of MSEL scales is significant, then the intercepts of the two scales are significantly different. We could then conclude that performance on a particular scale exhibits *delayed onset* in development relative to the other scale. If the interaction between chronological age and MSEL scales is significant, then we can conclude that the change in performance over developmental time was greater in one scale than the other, exhibiting a *faster rate* of development. In the case of the former, the profile would be consistent over time, while in the latter, it would alter.

## 3 Results

### 3.1 Background Information

#### 3.1.1 MSEL Descriptive Statistics

Table 2 presents the descriptive statistics for the Mullen Scales of Early Learning (MSEL). Both developmental quotient (DQ) and age equivalent (AE) scores are reported for each MSEL scale to provide context for interpreting the subsequent analyses and results.

**TABLE 2:** Descriptive statistics for MSEL scores at Time 1 (full cross‐sectional sample, *N* = 101/100 for visual reception) and Time 2 (longitudinal subsample only, *n* = 37).

		Time			
		Time 1 (full cross‐sectional sample)		Time 2 (longitudinal subsample only)	
		*M* *(* *SD* *)*	*Min*–*Max*	*M* *(*SD*)*	*Min*–*Max*
Chronological age (CA)		27.81 (13.34)	6.13–63.40	44.54 (7.98)	29.90–58.23
Developmental quotient (DQ)	Gross motor (GM)	52.32 (12.14)	29–89	47.23 (10.43)	19–64
	Fine motor (FM)	61.63 (13.70)	25–94	52.33 (10.31)	29–73
	Visual reception (VR)	63.06 (16.57)	30–102	59.97 (16.61)	32–109
	Receptive language (RL)	57.35 (18.07)	24–98	59.09 (15.96)	24–93
	Expressive language (EL)	55.86 (19.19)	16–126	47.07 (13.28)	30–98
Age equivalent (AE)	Gross motor (GM)	13.94 (6.21)	4–28	20.97 (5.46)	7–32
	Fine motor (FM)	16.36 (6.48)	3–30	22.97 (5.13)	16–42
	Visual reception (VR)	16.67 (7.79)	4–36	26.32 (6.88)	12–39
	Receptive language (RL)	15.76 (8.27)	2–36	25.97 (7.05)	9–41
	Expressive language (EL)	14.28 (6.68)	3–35	20.78 (6.15)	14–35

*Note:* CA and AE are in months; DQ = (AE/CA) × 100.

#### 3.1.2 DQ Scores Across Chronological Age

Although the focus of our paper is on analysing the DS profile utilising AE scores, as part of our background analyses, we examined the well‐documented decrease in DQ scores across chronological age (Grieco et al. 2015; Patterson et al. 2013). We ran a 5 × 2 mixed fully‐factorial ANCOVA with MSEL scales as the within‐subject factor and chronological age as a covariate. Although not a primary aim of the study, we followed the approach of others (e.g., Will and Roberts 2021) and checked for sex effects in these initial analyses (sex included as the between‐subject factor). There was a significant main effect of sex, *F*(1, 97) = 5.17, *p* = 0.025, η_p_^2^ = 0.05. Estimated marginal means showed that females scored significantly higher (*M* = 60.81, *SE* = 1.53) than males (*M* = 56.27, *SE* = 1.27). Chronological age was also significant, indicating that DQ scores decreased with age, *F*(1, 97) = 26.69, *p* < 0.001, η_p_^2^ = 0.22. Furthermore, there was also a significant main effect of MSEL scales, *F*(3.7, 357.4) = 6.70, *p* < 0.001, η_p_^2^ = 0.07 (Greenhouse–Geisser). This suggests an uneven profile across the MSEL scales, meaning that children's DQ scores differed significantly among domains. This unevenness did change significantly with age, *F*(3.7, 357.4) = 5.23, *p* < 0.001, η_p_^2^ = 0.05 (Greenhouse–Geisser). However, it did not significantly change with sex, *F*(3.7, 357.4) = 0.43, *p* = 0.772, η_p_^2^ < 0.01 (Greenhouse–Geisser). Therefore, for all the following analyses, we pooled across sex.

To further explore the effect of chronological age on MSEL individual scales, we tested the relationship of each scale and chronological age. Many of the scales show heteroskedasticity, with the language scales showing a greater increase in variability with age than the motor scales (for full analyses of variability, please see Results S1). Therefore, we employed the *vcovHC* argument (estimator HC3) in R (sandwich package 2.5.0; Zeileis et al. 2020) for the following regression analyses. This makes it possible to estimate heteroskedasticity‐consistent standard errors. Except for receptive language (*R* ^*2*^ < 0.01, *F*(1, 99) = 0.63; β = −0.08, *p* = 0.446), all scales showed a decrease in DQ with increasing age, *R* ^*2*^s > 0.11, *F*s(1, 98/99) > 13.00; βs < −0.34, *p*s < 0.001. In other words, with the exception of receptive language, the developmental progress of children with DS slowed as they got older. This pattern could reflect two possible effects: either the abilities of children with DS increase with age but at a slower pace (as is usually reported in the literature; for reviews, see Grieco et al. 2015; Patterson et al. 2013), or they show a true decline (regression) in abilities over time (Furley et al. 2023). To distinguish between these possibilities, in the next section we examine how age‐equivalent (AE) scores relate to chronological age.

#### 3.1.3 AE Scores Across Chronological Age

**FIGURE 2:**
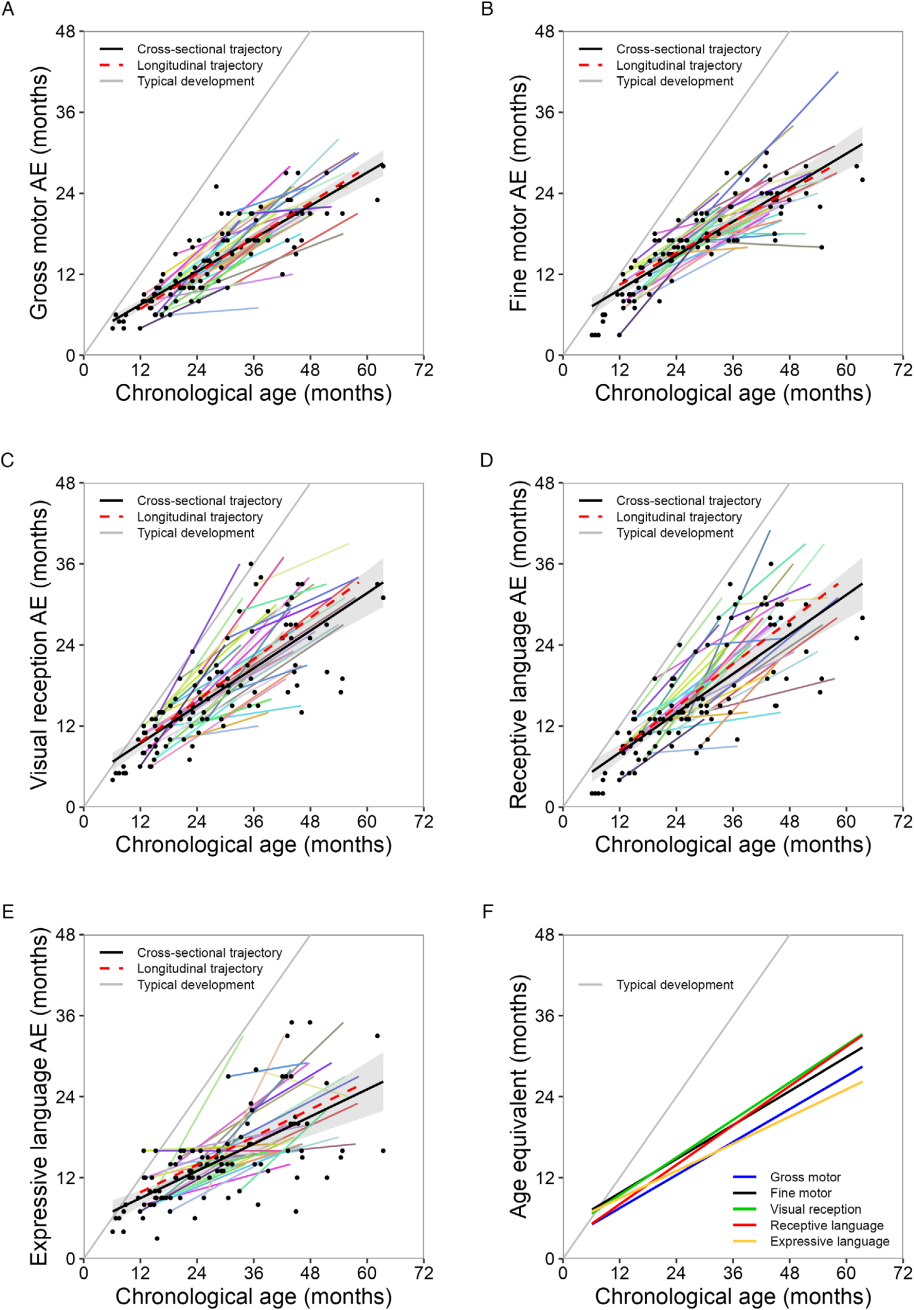
(A)‐(F) Each black dot corresponds to one participant with DS in a large cross‐sectional dataset that was used to construct cross‐sectional developmental trajectories (black regression lines with 95% confidence intervals in grey) of the relationships between chronological age and AE scores across five MSEL scales: (A) gross motor, (B) fine motor, (C) visual reception, (D) receptive language, (E) expressive language. A subset of the participants was followed up longitudinally, represented by coloured lines coming out of the relevant black dots. This longitudinal sample allowed us to construct longitudinal trajectories (red dashed lines). (F) Direct comparison of cross‐sectional developmental trajectories from five MSEL scales (blue = gross motor, black = fine motor, green = visual reception, red = receptive language, yellow = expressive language). For each figure, the grey line represents the average typical development trajectory for reference.

To understand changes in AE scores across chronological age, we plotted these two variables for each of the MSEL scales (Figure 2A–E). These graphs illustrate that as children with DS get older, they show an increase in their AE in all scales, *R* ^*2*^s > 0.44, *F*s(1, 98/99) > 80.70; βs > 0.67, *p*s < 0.001. To validate these cross‐sectional trajectories, we employed generalized estimating equations (GEE) with our longitudinal subset of data to generate longitudinal trajectories. The longitudinal trajectories were within the confidence intervals of the cross‐sectional trajectories (Figure 2A–E; the 95% confidence intervals of the cross‐sectional trajectories were computed based on heteroskedasticity‐consistent standard errors). This suggests that the cross‐sectional trajectories accurately capture and predict developmental change. Taken together, these analyses indicate that children with DS develop new abilities as they get older. However, with the exception of receptive language, they do so at a slower pace than the typical population (as indicated by the significant negative relationship of DQ and chronological age; see the section above) across domains.

### 3.2 Profile Analyses

#### 3.2.1 Cross‐Sectional Developmental Trajectory Analysis

In order to investigate changes in the MSEL profile across chronological age (Figure 2F), we employed a *cross‐sectional developmental trajectory analysis* (see Section 2.3). This analysis was carried out on square root transformed data to correct skew and kurtosis and with the covariate centred at the minimum chronological age of 6.13 months to test differences in intercept at the earliest age measured (the raw data are presented in the figures). There was a significant main effect of MSEL scales (*F*(3.5, 341.2) = 4.56, *p* = 0.002, η_p_^2^ = 0.04 [Greenhouse–Geisser]) indicating that the profile across the five scales of MSEL was uneven. Furthermore, there was a significant interaction between MSEL scales and chronological age (*F*(3.5, 341.2) = 5.82, *p* < 0.001, η_p_^2^ = 0.06 [Greenhouse–Geisser]) suggesting significant differences in how the scales changed across developmental time. In order to understand these changes across time, intercepts and slopes were compared for each pair of MSEL scales. Differences between scales are reported in Table 3.

**TABLE 3:** Partial correlations between AE scores from MSEL scales (accounting for chronological age; partial *r*) and comparisons of cross‐sectional developmental trajectories.

		GM	FM	VR	RL	EL
GM	Partial *r*	—				
	Intercept diff. (η_p_^2^)	—				
	Slope diff. (η_p_^2^)	—				
FM	Partial *r*	0.34***^B^	—			
	Intercept diff. (η_p_^2^)	0.11***	—			
	Slope diff. (η_p_^2^)	< 0.01	—			
VR	Partial *r*	0.34***^B^	0.52***^B^	—		
	Intercept diff. (η_p_^2^)	0.07**	< 0.01	—		
	Slope diff. (η_p_^2^)	< 0.01	< 0.01	—		
RL	Partial *r*	0.35***^B^	0.51***^B^	0.64***^B^	—	
	Intercept diff. (η_p_^2^)	< 0.01	0.09**	0.08**	—	
	Slope diff. (η_p_^2^)	0.03	0.05*	0.03	—	
EL	Partial *r*	0.08	0.44***^B^	0.31**^B^	0.48***^B^	—
	Intercept diff. (η_p_^2^)	0.05*	< 0.01	< 0.01	0.06*	—
	Slope diff. (η_p_^2^)	0.05*	0.07*	0.07**	0.15***	—

*Note:* A significant intercept difference indicates delay in one trajectory onset in relation to the other; a significant slope indicates a steeper or shallower developmental trajectory in one domain than the other. *p < 0.050; **p < 0.010; ***p < 0.001. For partial correlations; ^B^ *p* < 0.005 (Bonferroni corrected for 10 correlations).

Abbreviations: EL, expressive language; FM, fine motor; GM, gross motor; RL, receptive language; VR, visual reception.

As illustrated in Figure 2F and Table 3, the DS profile was uneven, and this unevenness changed over development. The gross motor scale differed significantly in intercept from the other scales (with the exception of receptive language) but not in slope (with the exception of expressive language), suggesting that, relatively speaking, gross motor development is an area of persistent weakness. Expressive language developed at a slower pace than the other domains, suggesting it is an emerging area of weakness. Even though receptive language had a lower intercept than the other scales (except for gross motor), from the lower initial level, it showed a relatively faster pace of development, particularly when compared with expressive language, indicating it is an emerging area of strength.

#### 3.2.2 Developmental Profile Before and After 24 Months

**FIGURE 3:**
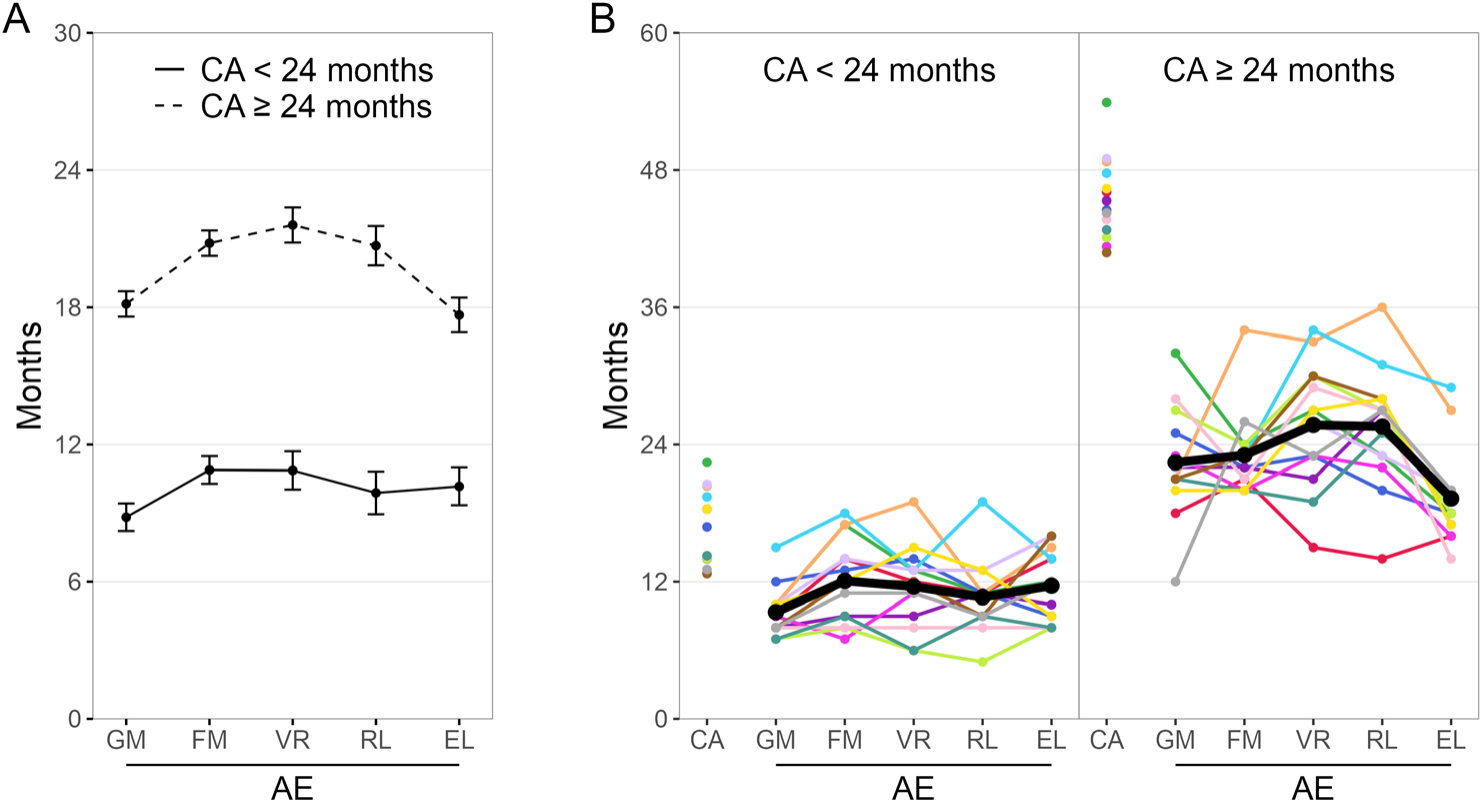
(A) Cross‐sectional profile in children with DS under 24 months (*n* = 46) and 24 months of age or older (*n* = 54). Error bars show ±1 SE. (B) Change in profile before and after 24 months of age in a small longitudinal sample (for details, see Results S2). Large individual differences (each coloured line corresponds to one child) in relative strengths and difficulties can be seen with a particular change in expressive language (thick black line corresponds to mean). AE, age equivalent; CA, chronological age; EL, expressive language AE; FM, fine motor AE; GM, gross motor AE; RL, receptive language AE; VR, visual reception AE.

Finally, we tested a suggestion from the literature that the profile in DS is established from around two years of age (Carr 1995; Cunningham 1996; Fidler et al. 2006; Marchal et al. 2016; Pezzuti et al. 2023). As Figure 3A illustrates, the profile in children younger than 24 months was indeed distinct from the profile in the older age group, with expressive language emerging as a second area of relative weakness alongside gross motor abilities (for full analyses, please see Results S2). Although it was too small a sample for statistical analyses, the longitudinal data is visually consistent (Figure 3B) with the finding from the larger cross‐sectional sample. However, as the profiles of individual children in Figure 3B illustrate, the emerging DS profile describes the average result in DS and is not applicable to every child with DS.

### 3.3 Concurrent and Longitudinal Associations Among Domains

As we saw in Figures 2 and 3, large individual differences exist among children with DS. To better understand these individual differences, we investigated associations across domains as well as the stability of individual differences across time. In our large cross‐sectional sample, we observed medium to large positive correlations between most of the scales after partialling out chronological age (see Table 3; the significance threshold after a Bonferroni correction was adjusted to 0.005). The only two scales which did not show a significant concurrent association were gross motor and expressive language. This suggests that children's level of difficulty in their gross motor abilities was not closely related to their expressive language abilities.

In terms of longitudinal associations, we found a large positive correlation for visual reception abilities (Table 4). This indicated that individual differences in visual reception were relatively stable over developmental time. No other associations met the significance threshold when a Bonferroni correction was applied (0.05/45), even though, with the exception of expressive language (0.11), the effect sizes ranged from 0.30 to 0.38. This suggests that the results have to be interpreted with caution as the reduced sample size also rendered most concurrent associations in the longitudinal sample non‐significant, despite having equivalent effect sizes to the cross‐sectional sample (see Tables 3 and 4).

**TABLE 4:** Partial correlations between MSEL scales in the longitudinal sample (*n* = 37) with two time points (Time 1 = T1, Time 2 = T2) accounting for the age at T1 and the gap between T1 and T2 (partial *r*).

		GM		FM		VR		RL		EL	
		T1	T2	T1	T2	T1	T2	T1	T2	T1	T2
GM	T1	—									
	T2	0.35*	—								
FM	T1	0.25	0.28	—							
	T2	−0.03	0.26	0.38*	—						
VR	T1	0.29	0.03	0.34*	0.41*	—					
	T2	0.07	0.25	0.33	0.49**	0.58***^B^	—				
RL	T1	0.50**	0.05	0.08	−0.12	0.52**^B^	0.25	—			
	T2	0.20	0.21	0.26	0.33	0.39*	0.64***^B^	0.30	—		
EL	T1	0.23	0.12	0.37*	0.16	0.26	0.18	0.39*	0.02	—	
	T2	0.14	−0.20	0.10	0.03	−0.01	0.26	0.25	0.52**^B^	0.11	—

*Note:* For the concurrent relationships within T1 and T2, the ages at T1 and T2 were partialled out respectively. *p < 0.050; **p < 0.010; ***p < 0.001; ^B^ *p* ≤ 0.001 (Bonferroni corrected for 45 correlations).

Abbreviations: EL, expressive language; FM, fine motor; GM, gross motor; RL, receptive language; VR, visual reception.

## 4 Discussion

### 4.1 DS Profile of Strengths and Weaknesses

In this study, we employed in‐depth phenotyping in a large sample with a wide age range to investigate how the profile of DS emerges over the first five years of life. Similarly to others (for reviews, see Grieco et al. 2015; Patterson et al. 2013), we observed a decrease in DQ of children with DS across development in most domains. In other words, although children with DS develop new abilities over developmental time (as indicated by an increase in AE scores), they do so at a *slower pace* than the TD population. This is perhaps a result of increasing developmental demands and the complexity of the abilities children are expected to learn. A slow pace of development was particularly pronounced for expressive language. As a result, this domain emerged as an area of relative weakness over developmental time. Gross motor development was also an area of relative weakness, persistently so. These patterns align with previous studies of the emerging DS profile (e.g., Dykens et al. 2006; Fidler et al. 2006; Pezzuti et al. 2023; Van Duijn et al. 2010; Will et al. 2018). Specifically, Fidler et al. (2006) highlighted relative weaknesses in expressive language and gross motor abilities at 2–3 years of age. Further, Dykens et al. (2006) identified expressive language as an area of relative difficulty in a broader sample ranging from one to 11.5 years. Our results are also in line with the profile described later in life in DS (Laws and Bishop 2003; Malak et al. 2015; Marchal et al. 2016; Martin et al. 2009; Miller and Leddy 1999; Sigman and Ruskin 1999; for recent reviews see Esbensen et al. 2024; Hamadelseed et al. 2023). Finally, we did not find a significant decrease in DQ with chronological age for receptive language. This is consistent with the existing literature which suggests that receptive language is an area of relative strength for individuals with DS (Abbeduto et al. 2007; Chapman and Hesketh 2000; Mason‐Apps et al. 2020; Miller 1999).

#### 4.1.1 Gross Motor Ability as Relative Weakness

We found gross motor ability to be an area of persistent relative weakness in the first five years of life. This is in line with previous studies reporting a number of early motor difficulties in DS, such as low muscle tone (hypotonia) and joint laxity (Block 1991; Cardoso et al. 2015; Cowie 1970; Lott 2012; Ulrich and Ulrich 1993), which are likely to constrain gross motor development. Indeed, others have reported a general delay in gross motor milestones in DS and this was most pronounced for later‐developing, more complex abilities (Cardoso et al. 2015; Malak et al. 2015; Palisano et al. 2001; Pereira et al. 2013; Tudella et al. 2011). For example, one early emerging motor skill, rolling, develops in many children with DS at a similar age to or just a couple of months behind TD children (Benjamin Neelon et al. 2012; Brouwer et al. 2020; Palisano et al. 2001; Pereira et al. 2013). Consider, in contrast, the later emerging motor skill of walking. While the average age of walking in TD children is 13 months and the age ranges from 9 to 17 months, most children with DS learn to walk between 18 and 36 months of age—with some children with DS unable to walk even at 4 years of age (Palisano et al. 2001; see also Winders et al. 2019).

Particular difficulties in the gross motor domain in DS may have a knock‐on effect on other domains as acquiring new gross motor abilities may alter the way young children interact with the world around them, changing their learning opportunities. For example, the transition from crawling to walking changes interactions between TD infants and their caregivers, increasing opportunities for more advanced social interactions (Clearfield et al. 2008; Karasik et al. 2011, 2014). This could potentially explain the positive relationship between walking and language development in young TD children (Walle and Campos 2014). Therefore, initially weaker gross motor abilities may constrain the development of other domains in DS. Even though the data from our smaller longitudinal sample do not show longitudinal correlations between gross motor abilities and other domains (larger longitudinal samples are needed to definitively test this hypothesis), in our larger cross‐sectional sample gross motor abilities were concurrently associated with fine motor, visual reception, and receptive language abilities. Similar associations were reported by Yamauchi et al. (2019) who observed posture‐motor abilities to be positively associated with both cognitive‐adaptive and language‐social abilities in 1‐ to 3‐year‐olds with DS. This association strengthened with increasing chronological age. Furthermore, onset of walking was positively associated with both cognitive and language development in these children with DS (Yamauchi et al. 2019). Finally, looking at a similar age range, Will and Roberts (2021) found gross motor abilities to relate to both expressive and receptive communication in DS. Longitudinal and intervention studies with large samples are needed to further probe possible developmental cascades involving gross motor abilities in DS. This seems to be an important focus of research, as young children with DS show particularly pronounced and persistent difficulties in gross motor development early in life.

#### 4.1.2 Receptive Language as an Emerging Relative Strength, Expressive Language as an Emerging Relative Weakness

We found receptive language to be an area of emerging relative strength. This is consistent with findings from other studies with older ages, smaller samples, and/or shallower phenotyping (Abbeduto et al. 2007; Chapman and Hesketh 2000; Mason‐Apps et al. 2020; Miller 1999). Over the first five years of life, the pace of development of receptive language did not show a decrease in DQ relative to chronological age (unlike the other domains). This contrasts with changes in expressive language, which became an area of relative weakness over developmental time due to a slower pace of development. Why do we observe such differences in these two language domains? To understand these differences, it is important to view these abilities developmentally, with challenges and demands changing over developmental time. To score high on the expressive language scale of the MSEL early in development, the infant needs to produce vocalisations, while early items on the MSEL receptive language scale require an understanding of meaning. The former may be easier for young children with DS to do than the latter. However, later expressive language items assess the ability to provide well‐formed spoken words and the ability to form high‐level concepts (Mullen 1995). Children with DS may find these developmental demands particularly challenging, which could lead to expressive language becoming an area of relative weakness as they get older. Indeed, infants with DS show a delay of around two months in the onset of canonical babbling compared to TD infants (Lynch et al. 1995). However, a delay of around 12 months has been reported for the ten‐word stage in children with DS (Oliver and Buckley 1994). As language complexity increases, so does the delay, with two‐word phrases showing a delay of around 18 months (Oliver and Buckley 1994). In other words, as children with DS develop, their understanding may improve more rapidly than the time it would take them to transition from vocalisations to words and to two‐word phrases.

The developmental differences in the language scales may be the result of the different number of factors constraining their development. Deckers et al. (2019) examined factors predicting variation in vocabulary development over a period of 1.6 years in 20 young children with DS between 2 and 7 years of age. Receptive vocabulary was best predicted by adaptive level of functioning and earlier receptive vocabulary, while many more variables were relevant for predicting expressive vocabulary (adaptive level of functioning, receptive vocabulary, level of maternal education, level of communicative intent of the child, attentional abilities, and phonological awareness). Indeed, other studies suggest that expressive language development in DS is a highly complex process contingent on multiple components, including nonverbal communication abilities, motor abilities, attentional abilities, face scanning, sleep, verbal short‐term memory, visuospatial short‐term memory, and family context (e.g., Chapman and Hesketh 2001; D'Souza et al. 2015; D'Souza, D'Souza, Horváth, et al. 2020; D'Souza, D'Souza, Jones, and Karmiloff‐Smith 2020; D'Souza, Lathan, et al. 2020; Deckers et al. 2019; Edgin et al. 2015; Martin et al. 2009; Mason‐Apps et al. 2018; Mundy et al. 1995). Compared to receptive language, then, different factors may contribute differently at different points in expressive language development. This could explain why we observed particularly low stability of individual differences (non‐significant correlation of 0.11) in expressive language across time. This is consistent with results from Sigman and Ruskin (1999) who also found that early expressive language did not predict later expressive language (non‐significant correlation of 0.23) in 61 young children with DS. However, they did find a correlation between early and late receptive language (correlation of 0.54), which in the current study did not reach significance (correlation of 0.30), perhaps due to a smaller sample size (37 children with DS). To detect the predicted correlation of 0.30, a sample size five times larger would have been required to reach the (Bonferroni adjusted) significance level of 0.05/45 (with 80% power). Interestingly, in our longitudinal subsample, the concurrent significant relationship between expressive and receptive language was found at Time 2 but not at Time 1. This perhaps suggests that the two domains become more intertwined over development. In our large cross‐sectional sample, expressive language was associated with receptive language, fine motor, and visual reception, suggesting that various domains constrain each other, and larger sample sizes may be necessary to investigate their relationships longitudinally.

### 4.2 Individual Differences Across Time

In line with our previous proposal (Karmiloff‐Smith et al. 2016), we observed large individual differences within each domain. Analysis of the residuals showed that the variability increased with age. In other words, according to the MSEL, young children with DS become more different from each other over time. Some caution needs to be exercised here as to whether this could be an artifact of MSEL measurement. However, there is support for our interpretation from a study including both DS and TD children. Albeit with a different tool (VABS), Van Duijn et al. (2010) investigated motor, daily living, communicative, and social behavioural skills in children with DS and TD children between birth and 12 years of age. Aligned with the current findings, in addition to a slower pace of development in these domains, the children with DS showed substantial dispersion around the regression line with increasing age compared to the TD children. If real, how could this increase in variability in DS across time be explained? We found this increase in variability to be higher for language scales than motor scales, potentially suggesting that the former is more developmentally malleable than the latter or that more diverse factors play a role here.

To investigate the stability of individual differences across time, we utilised our longitudinal subset of children with DS. This enabled us to examine the extent to which the abilities children show earlier in life are associated with later performance. We found stability only in visual reception abilities. This means that children who score lower on the visual reception scale early in development are likely to score lower in the same domain as they get older. There was no other significant longitudinal relationship in other domains. However, before we conclude that no other domain shows stable individual differences across time in this age group, it is necessary to consider how well powered our study was to detect these differences. As discussed above, it is possible that our longitudinal sample was too small (37 children) to detect significant associations. This is supported by contrasting the concurrent relationships between scales with differently sized samples. With our large cross‐sectional sample, all domains were concurrently related to each other (correlations ranged from 0.31 to 0.64) with the single exception of the relationship between expressive language and gross motor abilities (non‐significant correlation of 0.08). However, when the sample size was limited to the 37 children with DS followed longitudinally, only one concurrent association (between visual reception and receptive language) at Time 1 was found after applying a Bonferroni correction, even though the other correlation coefficients were as high as 0.50. As mentioned above, our power calculations suggested that a sample size at least five times that of the current longitudinal subset would be necessary to detect these effect sizes as significant.

### 4.3 Limitations

MSEL is widely used in the literature (Hahn et al. 2019; Levy et al. 2020). Compared to its alternatives (e.g., Bayley Scales of Infant and Toddler Development, Fourth Edition; Bayley and Aylward 2019), it covers a broader age range and has faster administration time. However, it is important to note that it has norms that are 30 years old (Mullen 1995). Furthermore, as with similar standardised tests, it has not been normed for neurodivergent children, such as children with DS, leading to floor effects for standard scores. To bypass this problem, AE scores are often used to highlight differences across domains or examine developmental delay (Toffalini et al. 2019), but it is necessary to consider their limitations as indicators of ability. As pointed out by Mullen (Mullen 1995), there are two main limitations to consider. Firstly, in the case of intellectual disability, children performing at a certain age may have learning and thinking abilities which are different from TD children of that same age. For example, a child with DS with an AE of 24 months in receptive language does not necessarily have the receptive language abilities of a TD 24‐month‐old. They may demonstrate a wide range of abilities, failing some tasks that TD children at 24 months are expected to pass, but succeeding at other tasks that TD 24‐month‐olds are not expected to successfully complete. Moreover, children with DS may be using abilities that are rarely present in TD children (e.g., Makaton), and these would not be captured by the standardised items of the MSEL. The second limitation relates to the comparison of AE scores across MSEL scales. The general measurement problem with AE scores is that their distribution is often different for each scale. Therefore, children who obtain the same standard scores across different scales may have slightly different AE scores. Thus, caution needs to be exercised when discussing individual children's profile of strengths and weaknesses, with more confidence when differences are large. Nevertheless, although these limitations of AE scores warrant the development of DS‐specific standardised tests for early years, which would be particularly important for any future clinical trials (Lee et al. 2020), AE scores provide a useful indication of performance at the group level.

Whilst maximising the inclusion of children with DS by utilising AE scores to avoid floor effects in the current study, some of the participants (3/104 in the full cross‐sectional sample; 6/43 in the longitudinal subsample) reached ceiling levels on one or more MSEL subscales. Although their exclusion was necessary to avoid biased estimates, it may limit the generalisability of our findings to individuals with DS who demonstrate developmental abilities beyond those captured by the MSEL.

Finally, it is also important to recognise that the MSEL provides only a snapshot of a child's abilities on a single day, which may not fully reflect their everyday functioning. Measures of day‐to‐day behaviour, such as parent‐reported adaptive functioning assessments (e.g., VABS; Sparrow et al. 1984), offer a valuable complementary perspective, as children's behaviours may differ day‐to‐day and across contexts (D'Souza and D'Souza 2024). At the same time, however, it is important to note that early in development, strong to very strong positive associations across individual domains (0.82–0.94; all *p*‐values < 0.001) have been reported between the MSEL and VABS‐3 in children with DS (La Valle et al. 2025). This suggests that, early in life, both measures may largely capture similar emerging milestones, resulting in substantial overlap in the abilities assessed. Future work would benefit from integrating adaptive functioning assessments alongside performance‐based measures to better characterise how these measures relate over developmental time.

### 4.4 Practical Implications

In order to provide children with DS with opportunities to flourish, it is crucial to make parents and practitioners aware of the uneven profile in DS and how it emerges over developmental time. This is particularly important as anecdotally we observed that during lab visits, parents (both of neurotypical and neurodivergent young children) often rely on two easily observable abilities—gross motor abilities and expressive language—as a proxy for overall developmental level. This may subsequently determine the choices of activities that the young child will be offered to engage in. Yet gross motor and expressive language are the two areas that were identified as relative difficulties in DS. Therefore, parents need to be aware that the use of these two domains to judge the overall developmental level of a child with DS may lead to an underestimation of abilities in other domains and potentially a lower level of engagement than would be optimal for that particular child.

Although we report on a general DS profile as it emerges over development, the profiles of the individual children with DS clearly show that no single profile can be assumed for all children with DS. In fact, we observed an increase in individual differences across age, particularly in the language domains. This is consistent with observations that children with DS follow diverse developmental trajectories rather than a uniform pathway (Karmiloff‐Smith et al. 2016). The growing variability highlights the importance of attending to within‐group heterogeneity when examining development in DS, as well as designing support activities that are responsive to the wide range of abilities and challenges within this population. Emerging literature on identifying subgroups in young children with DS (e.g., Fidler, Schworer, Prince, et al. 2019; Onnivello, Schworer, Prince, et al. 2023; Tsao and Kindelberger 2009) offers a promising direction for both advancing our understanding of DS development as well as providing individualised support. It is also important to be aware that the stability of individual differences may vary across domains. Here we observed relatively high stability of individual differences in visual reception. Therefore, it is likely that children with DS who show difficulties in visual reception abilities compared to their peers early in life will show difficulties in this domain later in life. In comparison, with the caveat of the small size of our longitudinal sample, individual differences in the other domains do not seem to be as stable. Thus, good performance in the other domains early in development may not necessarily mean that the child will perform well in these areas later on, as different abilities may be needed to come into play as the child develops. This emphasises a need for children with DS to be monitored across development rather than “signed‐off” when they show good progress early on.

## 5 Conclusion

In this study, we found that children with DS exhibit a slower rate of development as they get older, but this pattern is uneven across domains and age. While their early gross motor difficulties persist, their receptive language becomes an area of emerging relative strength, and their expressive language becomes a relative weakness. Variability increases with age, particularly in the language domains in comparison to the motor domains. With the exception of visual reception, we observed a lack of stability of individual differences in our longitudinal subsample.

Understanding how the DS profile emerges, as well as how stable the individual differences are, is important for practitioners and parents to ensure appropriate care and support for young children with DS.

## Funding

This work was supported by a Wellcome Trust Strategic Award (098330/Z/12/Z) conferred upon the London Down Syndrome (LonDownS) Consortium. For the purpose of open access, the authors have applied a CC BY public copyright licence to any Author Accepted Manuscript version arising from this submission. The work was also supported by the Waterloo Foundation, the Baily Thomas Charitable Fund, and the Jerome Lejeune Foundation. HD is a UK Research and Innovation (UKRI) Future Leaders Fellow (MR/X032922/1) and her work is further supported by the James S. McDonnell Foundation (JSMF; https://doi.org/10.37717/2022‐3711). The funding bodies had no role in study design, data collection, data analysis, data interpretation, writing of the report, or the decision to publish.

## Conflicts of Interest

The authors declare no conflicts of interest.

## Supporting information


**Results S1:** Variability.

**Results S2:** Differences in profile before and after two years of age.

## Data Availability

The study data are not publicly available due to ethical and privacy restrictions. The data that support the findings of this study are available from the corresponding author upon reasonable request, subject to appropriate approvals.
