# Supplementary material for: The Down Syndrome Profile Emerges Gradually Across Early Development
Source: J Appl Res Intellect Disabil. 2026 Jul 19;39(4):e70218. doi: 10.1111/jar.70218 (PMC13381579; doi:10.1111/jar.70218)
Supplement: Supplementary file 1 — Results S1: Variability. [file JAR-39-e70218-s002.docx]

## Results S1: Variability

Visual inspection of Figure 2A–E (main text) suggests that variability increases over developmental time in DS (i.e., heteroskedasticity) and this increase seems uneven across domains. Before we consider the origin of this developmental change, whether it represents true ability levels or measurement issues, let us first establish its statistical reliability. We extracted absolute values of residuals from linear regressions predicting AE from chronological age. As many of the variables showed heteroskedasticity, we employed the *vcovHC* argument (estimator HC3) in R (sandwich package 2.5.0; Zeileis et al., 2020) for the following regression analyses. Indeed, as children with DS get older, they tend to show an increase in their residuals (trend in fine motor: *R^2^* = .05, *F*(1, 99) = 4.84; β = .22, *p* = .093; significant effect for other scales: *R^2^s* > .06, *F*s(1, 98/99) > 6.76; βs > .25, *p*s < .012). To test whether the increase in variability was significantly different across scales, we conducted a mixed ANCOVA with MSEL scales (five scales) as a within-subject factor and chronological age as a covariate. This analysis was carried out on square root transformed data to correct skew and kurtosis and with the covariate centred at the minimum chronological age of 6.13 months (the raw data are presented in the figures [main text]). The main effect of MSEL scales was not significant, *F*(4, 392) = 1.40, *p* = .234, η_p_^2^ = .01. However, there was a significant interaction between MSEL scales and chronological age (*F*(4, 392) = 4.65, *p* = .001, η_p_^2^ = .05), suggesting significant differences in the way in which variability changed across developmental time. In order to understand these changes across time, intercepts and slopes were compared for each pair of MSEL scales. Differences between scales are reported in Table S1. As illustrated in Figure 2A–E (main text) and Table S1, changes in variability were not uniform. Notably, the language scales showed a greater increase in variability with age than the motor scales as indicated by significantly different slopes.

Table S1

*Comparisons of cross-sectional developmental trajectories of variability in MSEL scales (absolute values of residuals from linear regressions predicting AE in the respective scale from chronological age). A significant intercept difference indicates delay in one trajectory onset in relation to the other; a significant slope difference indicates a steeper or shallower developmental trajectory in one domain than the other.*

*
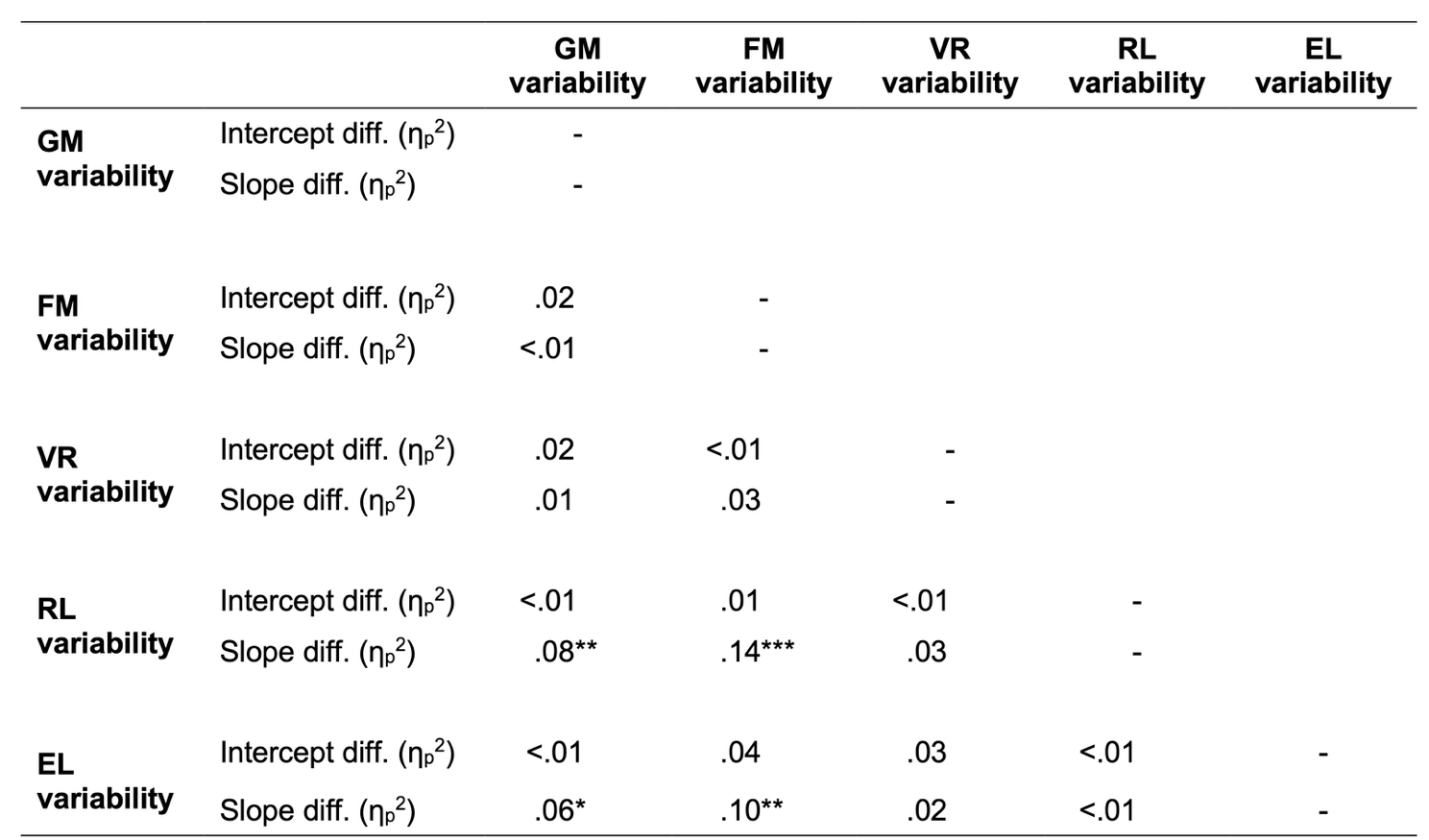
*

*Note*. GM = gross motor, FM = fine motor, VR = visual reception, RL = receptive language, EL = expressive language; **p* < .050, ***p* < .010, ****p* < .001.
