# Supplementary material for: The Down Syndrome Profile Emerges Gradually Across Early Development
Source: J Appl Res Intellect Disabil. 2026 Jul 19;39(4):e70218. doi: 10.1111/jar.70218 (PMC13381579; doi:10.1111/jar.70218)
Supplement: Supplementary file 2 — Results S2: Differences in profile before and after two years of age. [file JAR-39-e70218-s001.docx]

## Results S2: Differences in profile before and after two years of age

Based on Figure 2F (main text), the relative position of expressive language to other domains changed over development. It has been suggested in the literature that the individual differences in DS show some degree of stability from around two years of age (Carr, 1995; Cunningham, 1996; Fidler et al., 2006; Marchal et al., 2016; Pezzuti et al., 2023). To test this idea, we divided our participants from our large cross-sectional sample into those who were younger than 24 months and those who were 24 months of age and above. Age was now modelled as a categorical variable instead of a continuous variable. A 5 x 2 mixed ANOVA was employed with MSEL scales as the within-subject factor and age group as the between-subject factor. This analysis was carried out on square root transformed data to correct skew and kurtosis, and improve homogeneity of variances (the raw data are presented in the figures [main text]). As would be expected, there was a significant main effect of age group (*F*(1, 98) = 134.81, *p* < .001, η_p_^2^ = .58) with the older group having higher AE scores than the younger group. This applied across all scales, *t*s(98/99) > 7.27, *p*s < .001, *d*s > 1.45. There was also a significant main effect of MSEL scales, *F*(3.5, 340.7) = 12.46, *p* < .001, η_p_^2^ = .11 (Greenhouse-Geisser). This reiterates the uneven profile across the MSEL scales. Crucially, the uneven profile differed across the two age groups as indicated by the MSEL scale and age group interaction, *F*(3.5, 340.7) = 3.81, *p* = .007, η_p_^2^ = .04 (Greenhouse-Geisser).

Figure 3A (main text) illustrates that the profile in the younger age group was distinct from the profile in the older age group. At a younger age, children showed a relative weakness in gross motor abilities as indicated by paired sample *t*-tests, which showed a significant difference between the gross motor scale and the other scales (with the exception of receptive language), *t*s(45) > 2.49, *p*s < .017, *d*s > 0.36. Similarly, in the older age group, gross motor remained low relative to the other scales (with the exception of expressive language), *t*s(53/54) > 2.54, *p*s < .014, *d*s > 0.34. As with the cross-sectional analysis presented in the main text, this suggests that across development, gross motor presents a persistent area of relative weakness (in fact, for 37% of the children under 24 months and 33% of the children at or above 24 months, gross motor was their lowest score across the scales). In line with the cross-sectional trajectory analyses presented in the main text, the most marked change across the two age groups was present in expressive language. While expressive language did not significantly differ from fine motor, visual reception, or receptive language in the younger age group (*t*s(45) < 1.19, *p*s > .241, *d*s < 0.18), it did in the older one (*t*s(53/54) > 3.45, *p*s < .002, *d*s > 0.46). In fact, in the older age group, expressive language was on the same level of relative weakness as gross motor, *t*(54) = 0.85, *p* = .402, *d* = 0.11. Thus, from 24 months, expressive language becomes an area of relative weakness (13% of children under 24 months vs. 38% of children at or above 24 months scoring lowest in this scale) alongside gross motor abilities.

To confirm the above pattern of results, we examined data from a smaller longitudinal sample. We selected those children who we followed longitudinally and who were younger than 24 months at Time 1 and 24 months or older at Time 2 (thus mapping onto the age groups above). From these children, we selected only those who belonged to the 30-month gap between Time 1 and Time 2 (to allow for enough time for longitudinal change). Fourteen children fit these criteria. It is important to emphasize that the Time 1 data from these 14 children also belong to the under 24 months group in the larger cross-sectional sample above. Therefore, we are primarily interested in the Time 2 data as these are independent of the 24 months and up group. Although the sample was too small for statistical analysis, the longitudinal data is visually consistent (Figure 3B, main text) with the finding from the larger sample (Figure 3A, main text). Whereas visually gross motor abilities presented an area of relative weakness across both ages, expressive language only became an area of difficulty when the children were older. However, as profiles of individual children in Figure 3B (main text) illustrate, the emerging DS profile describes an average result in DS and is not applicable to every child with DS.

Overall, analyses with chronological age as a categorical variable confirm, and make more salient, the altering profile observed in the children with DS.
